# Analysis of Movement and Actions of Wingers as Second-Line Players in Organized Attack in Handball

**DOI:** 10.5114/jhk/175281

**Published:** 2024-02-17

**Authors:** Dimitris Hatzimanouil, Jose M. Saavedra, Afroditi Lola, Vasilis Skandalis, Konstantinos Gkagkanas

**Affiliations:** 1 School of Physical Education and Sports Science, Aristotle University of Thessaloniki, Thessaloniki, Greece.; 2 Physical Activity, Physical Education, Sport and Health (PAPESH) Research Centre, Sports Science Department, School of Social Sciences, Reykjavik, Iceland.

**Keywords:** modern handball, run-in, pivot, correspondence analysis

## Abstract

In modern handball, one of the important performance indicators is the effectiveness of the attack, especially the running-in of wingers as line players which has not been explored adequately. The purpose of the study was to analyze the movements of wingers in the organized attack when they run in. Fifty-eight matches were analyzed from the 2022 EHF European Men’s Handball Championship. A total of 491 attacks were recorded and 45 variables were analyzed in which wingers ran in as second pivots and the outcome was a throw. For the statistical analysis, descriptive and inductive statistics were used. The results showed that the average time of the running-in was 12.11 ± 9.28 s, the left wing ran in more often (60.1%) and wingers tended to move outside the defense formation (72.5%). Wingers ran in without the ball in possession (81.1%), moved toward defenders 2 and 5 and stood next to defenders, and occasionally blocked, slid or left their position. The defense’s central zone was preferred by players to make a throw. Correspondence analysis showed that wingers finally returned to their initial position regardless of the evolution of the attack. Their role was to block a specific defender or disorganize the defense, for one defender to be isolated so that one attacking player could execute a shot from the central area. Conclusively, wingers play an important role, especially in run-in actions, at the completion of an attack, and in the final throw.

## Introduction

The determination of performance indicators in handball as a research field has developed rapidly in recent decades and provided coaches with a lot of useful information (Cabrera Quercini et al., 2022). According to Srhoj and colleagues (2001), the outcome of a match is the product of the interaction of the two opposing teams manifested through game elements and external environmental influences. Those elements that have the greatest influence on the result are identified as performance indicators. In essence, performance indicators are a selection or combination of variables from actions that tend to determine some or all aspects of performance. Furthermore, cognitive traits such as concentration, peripheral vision, short-term memory and reaction time play an essential role in performance (Blecharz et al., 2022).

In recent years efforts have been made to identify the best performance indicators in handball. These have led to some agreement on the importance of certain variables that allow separation and discrimination between winning and losing teams (Beiztegui-Casado et al., 2019; Mikołajec et al., 2021; Saavedra et al., 2017). Counterattacks, shots from the 6-m line (close throws), and goalkeeper involvement are the performance indicators that appear most frequently in the studies reviewed (Cabrera Quercini et al., 2022). New forms of analysis have recently been introduced (Russomanno et al., 2021). These are mainly based on systematic observation and are called semiotic analysis in the field of sports science (Anguera and Hernández Mendo, 2013).

However, even though, as mentioned by Cabrera Quercini et al. (2022), one of the important performance indicators is the effectiveness of the attack, individual actions that lead to an effective organized attack have not been thoroughly analyzed and clarified. An important and frequent action in the organized attack is the running-in of wingers into 6 meters as second-line players. Georgiana and Aurelia (2014) state that considering the tasks of the winger's position during the game, modern European handball requires a lot of involvement of these wingers in the tactics of the game. According to the same authors, who analyzed the 2012 European women's championship, the average of winger goals in that tournament was 6.1 goals per match. In total, these players scored 668 goals. Of these 51 were from the position they ran in as a second line player, that means 7.63% of the total goals scored by these players. In addition, those authors state that regarding the lateral position (wing) in which the wingers play, this is often the position from which other players who are not specialized in that particular position and are not wingers make an effort to score goals.

Therefore, we can conclude that wingers participate in technical-tactical actions (leaving their position and having moved into free space on the 6-m line), which create situations for the goals to be scored by their teammates. The above authors conclude that the participation of wingers in technical-tactical actions of their teams varies from team to team, depending on the school of handball from which each team comes, but also depending on the individual value of players who specialize in this particular position. The second position that the wingers specialized in and played in this tournament was that of a line player (as a second pivot), with movements inside the 6-m line with or without the ball, their specific actions resulting in their teammates scoring goals. Wingers significantly improved their movements without the ball, since during attacks the winger does not have possession of the ball most of the time (Georgiana and Aurelia, 2014).

Therefore, these specific players can contribute to the effectiveness of an organized attack by their actions, either scoring themselves or allowing their teammates to score, and contribute to the performance index concerning the effectiveness of the attack. As Anton-Garcia (2011) mentions, the success of a team also depends on the activity of players without the ball, whose actions facilitate the game of their teammates.

Although some performance indicators have been adequately analyzed, the analysis of wingers’ movements when they run in 6 meters as second pivots has not been explored. This led to the purpose of the present study, which was the analysis of the movements of wingers in the organized attack, and more specifically, when they moved from their basic position and ran in 6 m as second pivots until the result and the final outcome of the specific phase of the attack when it resulted in a throw.

## Methods

### Participants

Fifty-eight matches were analyzed from the 2022 European Handball Federation (EHF) Men’s Handball Championship which took place from the 13^th^ to the 30^th^ January, 2022, in Hungary and Slovakia. A total of 491 attacks were recorded in which wingers ran into 6 meters as second pivots and the outcome of the attack was a throw. In cases where the winger ran into 6 meters as a second pivot, but no throw was made at the end of the attack (technical errors, passive game, ball steals by the opponent), these attacks were not analyzed further and were not included in the analysis of the present study.

The Research Ethics Committee of the School of Physical Education and Sport Science in Thessaloniki approved the design of that study (approval code: 160/2023; approval date: 28 June 2023), because it fulfills the criteria of good scientific practice, as set out in the Regulation of Principles and Operation of the Research Ethics Committee of the Aristotle University of Thessaloniki and in the existing legislation.

### Measures

A total of 45 variables were recorded and analyzed (Table 1).

**Table 1:** Studied variables.

Name	Description
Time (s)	Duration of an attack from 2 to 65 s
Initial attack position	Left, right
Direction of movement	Inside from 6 m or outside
Ball possession	Yes, No
Final attack position	One, two, three, four, five, six
Action without the ball	No action, block, stay in the position, sliding, leave
Type of a block	No block, back block, lateral block, front block, forward move block, backward move block
Reception of the ball	Yes, No
Receive foul	Yes, No
After foul	Keep the position, back to the initial position
Action with the ball	Deblock gets the ball and throws, throws without deblocking, gets the ball and passes, gets the ball and fouls, gets the ball and wins penalty
**1^st^ “slide”**	
Ball possession	With or without
Defender position	Defender no. 1 (left-right), def. 2 (left-right), def. 3 (left-right), def. 4 (left-right), def. 5 (left-right), def. 6 (left-right)
Action	No action, block, stays in the position, “slides”, returns to the initial position
Type of a block	No block, back block, lateral block, front block, forward move block, backward move block
Reception of the ball	Yes, No
Receive foul	Yes, No
After foul	Keep the position, back to the initial position
Action with the ball	Deblock gets the ball and throws, throws without deblocking, gets the ball and passes, gets the ball and fouls, gets the ball and wins penalty
**2^nd^ “slide”**	
Ball possession	With or without
Defender position	Defender no. 1 (left-right), def. 2 (left-right), def. 3 (left-right), def. 4 (left-right), def. 5 (left-right), def. 6 (left-right)
Action	No action, block, stays in the position, “slides”, returns to the initial position
Type of a block	No block, back block, lateral block, front block, forward move block, backward move block
Reception of the ball	Yes, No
Receive foul	Yes, No
After foul	Keep the position, back to the initial position
Action with the ball	Deblock gets the ball and throws, throws without deblocking, gets the ball and passes, gets the ball and fouls, gets the ball and wins penalty
**3^rd^ “slide”**	
Ball possession	With or without
Defender position	Defender no. 1 (left-right), def. 2 (left-right), def. 3 (left-right), def. 4 (left-right), def. 5 (left-right), def. 6 (left-right)
Action	No action, block, stays in the position, “slides”, returns to the initial position
Type of a block	No block, back block, lateral block, front block, forward move block, backward move block
Reception of the ball	Yes, No
Receive foul	Yes, No
After foul	Keep the position, back to the initial position
Action with the ball	Deblock gets the ball and throws, throws without deblocking, gets the ball and passes, gets the ball and fouls, gets the ball and wins penalty
Final position of the shot	Left winger, right winger, left back, right back, center player, pivot
Final attempt	Left winger, right winger, left back, right back, center player, pivot
Outcome	Goal, out, post, save

### Design and Procedures

The recording and analysis of the attacks were performed by two handball experts in coaching and training. In cases where these two experts’ assessment analysis differed, two further experts analyzed the same game situation. If fewer than three experts were assigned to the analysis of a specific game situation, it was considered unclassified and not taken into account.

### Statistical Analysis

Descriptive and inductive statistics were used in the present study. More specifically, the frequency of the values and their corresponding percentage, as well as the mean value and standard deviation (SD) were used. Correspondence analysis was also applied in order to verify the differentiation of wingers, in terms of their movement duration and their movement/ action content on the one hand and their ball possession on the other. Furthermore, correspondence analysis was applied in order to verify the differentiation of wingers, in terms of the final position and the player position of the final attempt. A *p*-value of <0.05 was considered statistically significant. The statistical analysis was performed with the software package IBM SPSS Statistics for Windows, Version 25.0 (IBM Corp. Released 2017. Armonk, NY: IBM Corp.).

## Results

Descriptive statistics showed that the average time duration of the running-in of the winger as the second pivot was 12.11 ± 9.28 s to the final conclusion of the attack and the final throw. In the 491 cases analyzed, left wingers ran-in 295 times (60.1%), while right wingers ran in 196 times (39.9%). Furthermore, results showed that on 356 occasions (72.5%), when a winger ran in, they moved outside the defense formation and then entered the 6-m line. On the contrary, the player moved internally, through the defense formation, 135 times (27.5%). The winger ran in without having the ball in possession 398 times (81.1%), while they had the ball in their possession 93 times (18.9%). In terms of the position of the defender that the winger ran into, the results showed that wingers most often moved toward defenders 2 and 5 when they were positioned either to the left or the right of these defenders (Table 2).

**Table 2:** Position of the defense the winger runs into either on the right or the left side of the defender.

Defender’s position: Number	Frequency	Percentage
1	43	8.8
2	109	22.2
3	86	17.5
4	86	17.5
5	94	19.1
6	73	14.9
Total	491	100

The results also showed that when a winger ran in, their action was most often (63.7%) staying and standing next to the defender. On fewer occasions (19.6%), the winger blocked, left the position they had moved to (10.8%), or slid (5.9%). When the winger blocked, in 46% of cases it was with the back, 8% laterally with the shoulder, 32% with the chest, 10% forwards, and 5% backwards. In 90.8% of cases, wingers did not receive the ball when they were on the 6-m line, while in 9.2% they received the ball. Furthermore, the results of this study showed that 98.0% of attacks resulting in a final throw were completed without a foul to the winger or another player. Finally, in the cases where the winger received the ball, they received the ball with deblocking and made a throw 4 times (13%), they received the ball without deblocking and made a throw 22 times (66%), they received the ball and made a pass 3 times (8%) and they received the ball and won a penalty 4 times (13%).

Table 3 shows the attacking position and the shooting player for the final throw and the culmination of all team action from the beginning to the end. Table 4 shows the outcome of the final attempt.

**Table 3:** Attacking position of the shot and the shooting player.

	Attacking position		Shooting player	
Attacking position	Frequency	Percentage	Frequency	Percentage
Left wing	38	7.7	41	8.3
Left back	91	18.5	111	22.6
Central player	157	32.0	127	25.9
Right back	69	14.1	83	16.9
Right wing	38	7.7	51	10.4
Pivot	98	20.0	78	15.9
Total	491	100%	491	100%

**Table 4:** Outcome of the final attempt.

Outcome	Frequency	Percentage
Goal	287	58.5
Out of the goal	51	10.4
Save	130	26.5
Post	23	4.6
Total	491	100%

**Figure 1:**
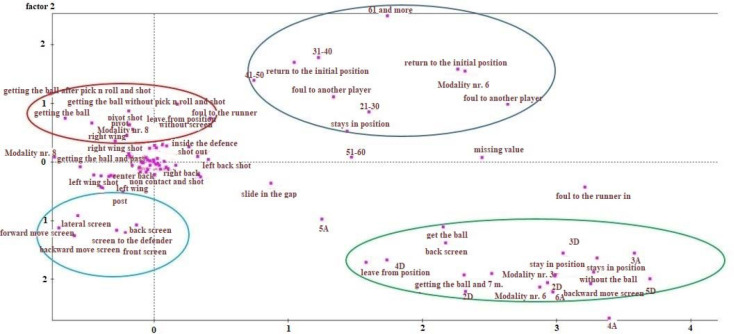
Correspondence analysis from the 1X2 horizontal and vertical axis showing how the total of attacks is separated and grouped.

The 1X2 correspondence analysis (Figure 1) found that the total of attacks was divided into two groups (horizontal axis): a) attacks with a winger as a second line player who made a block to other defenders at the line and stayed in the position without being fouled and without receiving the ball, and b) attacks with a winger as a second line player who was in defense and then left, returning to their initial position, or received the ball, sustaining a possible foul, and again returned to their initial position. Thus, the attack evolved in two ways: either the player did not participate after entering and then left via the space of initial entry, or they participated after entering by taking the ball with the possibility of being fouled, and finally returning to their initial position. Thus, the main outcome was that the player finally returned to their initial position regardless of the evolution of the attack. The correspondence analysis also showed that the total of attacks was divided into two further groups (vertical axis): a) attacks of long duration with a winger running in as a second-line player, and b) attacks of short duration with a winger running in as a second-line player. The attacks were also grouped along the vertical and horizontal axes into those in which the winger entering as a second-line player took the ball and after passing either left or stayed (Figure 1, top left); attacks where they blocked (Figure 1, bottom left); attacks of long duration (more than ten seconds) (Figure 1, top right); and finally attacks where the winger entering as a second line player “slid” and stayed, without taking the ball (Figure 1, bottom right).

**Figure 2:**
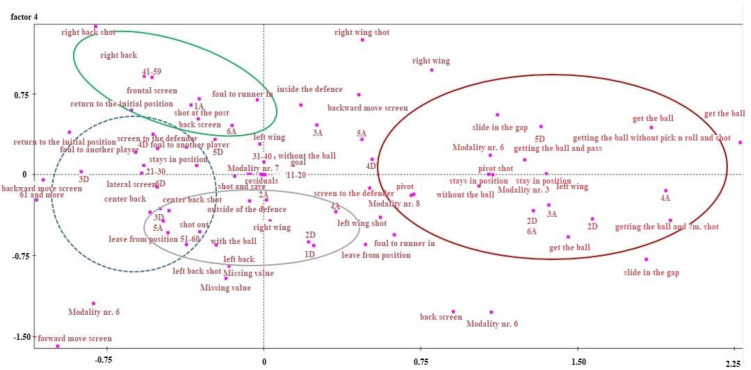
Correspondence analysis from 3X4 horizontal and vertical axis showing how the total of attacks is separated and grouped.

The third axis of the 3X4 correspondence analysis shows that the total of attacks was divided into two groups: a) those in which the winger received the ball and those in which they did not. The fourth axis of the correspondence analysis shows that the total of attacks was grouped according to the position from which the final attempt was made and the player who took the shot. The attacks were also grouped along the vertical and horizontal axes into those in which the winger entered without the ball and either blocked (back, chest, front) or returned to their initial position, and those in which the entering winger took the ball while they were inside and acted. Also, in the same situation, when the player did not receive the ball, they executed a “slide” (without the ball). The final shot in this situation was from the pivot position and the player who took the shot was either a pivot or a left wing. Another situation was when left wingers entered as a second-line player, and took the position left by the number one defender. In situations where they did not receive the ball, the final attack was from the right back or the right-wing position, and the shot was executed by the right back or the right wing player. Finally, another case is when the player entered as a second-line player (they were the right wingers who went left, e.g. to the right side of defender number one or the right side of defender number two), and in the attacks when they did receive the ball, the final attack was made by the left back and center back positions, while the shot was executed by the left back or the center back player.

## Discussion

The findings of this study provide insights into the running-in patterns of wingers. The average time duration of the running-in was 12.11 ± 9.28 s, indicating that wingers play an important role in the final conclusion of an attack and the final throw. These results are consistent with previous studies that have highlighted the crucial role of wingers in handball games, especially in run-in actions (Karcher and Buchheit, 2014). The results also showed that left-wing players ran in more often (60.1%) than players at the right-wing position (39.9%). This finding is consistent with the handedness of most players, as the majority of handball players are right-handed. However, handedness is not crucial when it comes to scoring in penalty situations (Laxdal et al., 2022).

Furthermore, the results indicated that wingers tended to move outside the defense formation when running in (72.5%) rather than internally through the defense formation (27.5%). This may be due to the fact that wingers can take advantage of the space outside the defense formation and create scoring opportunities. Interestingly, wingers in this study mostly ran in without having the ball in possession (81.1%). This finding suggests that wingers may be used more frequently as decoys or to create space for other players rather than as primary ball carriers. In this regard, a study conducted on the Croatian league suggests that winning teams are clearly characterized by quick attacks against a disorganized defense (Rogulj et al., 2004). On the other hand, the results showed that wingers mostly moved towards defenders 2 and 5 (Table 2), positioned either to the left or the right of these defenders.

Regarding the position, the study found that wingers most often stayed and stood next to defenders, and only occasionally blocked, slid, or left their position. When they did block, it was mostly with their chest or laterally with their shoulder. Additionally, wingers were rarely found to receive the ball when they were on the 6-m line. This seems to indicate that coaches could encourage wingers to be more aggressive in their blocking and to move more frequently to different positions on the court to increase their chances of receiving the ball. The preferred position for players to make the throw is the central zone (Table 3), which seems logical as this area allows for a more centered angle to execute a shot. In addition, it should be noted that these players (back players) are those who achieve the highest throwing velocity (Foretić et al., 2021). Similarly, there is a higher tendency for second-line players to finish the play, with the final goal percentage being above 58% (Table 4). However, a higher team ranking was associated with higher throwing efficiency, but only for wingers (Pueo et al., 2023).

From the results of the 1X2 correspondence analysis, it seems that the main outcome is that after wingers run in the defense as a second line player, they finally return to their initial position regardless of the evolution of the attack. In modern handball this is happening because it is difficult for the winger as a second-line player to receive the ball considering that defensive formations are very compact nowadays (Spiridon, 2014). Consequently, their main role is a) to block a specific defender when they run in, as Spiridon (2014) states, or b) to disorganize the defense by returning to their initial position in order for one defender to be found isolated without any support from the other defenders (Georgiana and Aurelia, 2014; Prisicaru, 2015).

The correspondence analysis results also showed that the total of attacks is divided into two groups (vertical axis): a) attacks of long duration with a winger running in as a second-line player, and b) attacks of short duration with a winger running in as a second-line player. This makes sense because of the different evolution and the final outcome of the attack, due to the fact that attacks are temporarily interrupted by faults (long duration) or completed in a very short time (short duration) (Guignard et al., 2022).

The vertical and horizontal axes also show that the attacks can be divided into the following groups: A) Those in which the winger who enters as a second-line player takes the ball and after passing either leaves or stays (top left in Figure 1). This appears to happen because it is very difficult for wingers to break through with the ball due to the compactness of the defenses, so they pass the ball and then act in various ways (Spiridon, 2014). B) Attacks where the winger blocks (bottom left), either in order to receive the ball after the block or to block a defender. C) Attacks of long duration (more than ten seconds) (top right in Figure 1). This is because defenses adapt and try to make faults and interrupt the evolution of the attack early (Guignard et al., 2022). Finally, D) Attacks where the winger enters as a second-line player, “slides” and stays without taking the ball (bottom right in Figure 1). This is probably due to a) attack of long duration, and b) advanced defense movements, so the second-line player finds free spaces to move in and “slide” into the gaps of the defense (Georgiana and Aurelia, 2014).

The 3X4 correspondence analysis shows that the total of attacks can be divided into two groups: a) those in which the winger receives the ball, and b) those in which they do not. The attack thus evolves depending on the team tactics (Georgiana and Aurelia, 2014). The fourth axis of the correspondence analysis shows that the total of attacks is grouped according to the position from which the final attempt is made and the player who takes the shot. This makes sense because it seems that the tactical attack of a team differentiates the final attempt, which is defined by the space of the final attempt and the attacking player’s position (Hatzimanouil, 2019; Hatzimanouil et al., 2017).

The attacks are also grouped along the vertical and horizontal axes into those in which the winger enters without the ball, and either blocks (back, chest, front) or returns to their initial position. Another case is when the winger who is going inside, takes the ball while inside and acts, or when the player does not receive the ball and executes a “slide” (without the ball). The final shot in this situation is from the pivot position and the player who takes the shot is either the pivot or the left winger. It is clear that the tactical choice depends on whether the second-line player receives the ball or not. Often the final attempt is executed from the 6-m line due to the presence of two line players (a pivot and a winger as a second pivot) (Sibral, 2014). Another situation is when those entering as a second-line player (these are left wingers and go to a position left of defender number one) do not receive the ball, and the final attack is from the right back or the right wing position, with the shot being executed by the right back or the right wing player. This happens because the left side of the defense is overloaded, outnumbering the attack on the right side of the defense (Varzaru et al., 2019).

Finally, another case is when wingers enter as a second-line player (right wingers who go left, e.g., to the right side of defender number one or the right side of defender number two), and in the attacks when they do receive the ball, the final attack is made by the left back and center back positions, while the shot is executed by the left back or center back players. In the above cases, when right wingers receive the ball in their movement, after passing it they block defenders 1 and 2 with their entry in order to overload the defense in this attacking zone while restricting the exit of defenders. The ultimate goal is for the final attempt to be made under good conditions by the left-back or the center-back player (Varzaru et al., 2019).

Although the strong characteristic of our study is the high level of players and competition that comprised the sample, the results should be considered with caution and few limitations need to be taken into account. The first limitation is that the sample of the present study refers to high-level players and competition. Although there was a high number of games, another limitation is that such a high-level competition as the European Men’s Handball Championship lasts for a small amount of time. There is a need for future research over a longer period. The third limitation is that the study sample consisted only of men. In future research, the female population could also be included. The fourth limitation involves the lack of past research on this specific subject regarding handball. Therefore, our study aims to fill this blank in the existing literature. Clearly, given these limitations, there is a need for more research on this subject in the future, to fully make clear this specific subject of handball tactics.

## Conclusions

To conclude, we would say that wingers play an important role, especially in run-in action, in the final conclusion of an attack, and in the final throw. Left-wingers ran in more often than right-wingers, tending to move outside the defensive formation when running in rather than internally through the defense formation, and they mostly ran in without having the ball in possession. Wingers moved towards defenders 2 and 5 and positioned themselves either to the left or the right of these defenders. They stayed and stood next to these defenders, and only occasionally blocked, slid, or left their position. They also rarely received the ball when they were on the 6-m line. Thus, wingers were used as decoys or to create space for other players rather than as primary ball carriers. This was also established by the correspondence analysis, which showed that after running-in the defense as a second-line player, wingers finally returned to their initial position regardless of the evolution of the long- or the short-duration attack. Consequently, their main role was to block a specific defender when they ran in or to disorganize the defense by returning to their initial position in order for one defender to be found isolated without any kind of help from the other defenders. Furthermore, the correspondence analysis also showed that the attacks were grouped into those in which the winger received the ball and those in which they did not. In these two situations, the main role of wingers was to overload one part of the defense by entering, while restricting the exit of the next defenders, so that one attacking player, usually the center or the left- and the right-back player, could execute a shot. Therefore, wingers’ game, especially in run-in action, demonstrates a variety of movements of which the purpose is to contribute to the effectiveness of an organized attack. It is obvious that more data are needed to establish the modern way of playing the wingers’ game when they run in as a second-line player.
